# Surgical Capacity in Public and Private Health Facilities After a Five-Year Strategic Plan Implementation in Ethiopia: A Cross Sectional Study

**DOI:** 10.5334/aogh.3871

**Published:** 2023-03-09

**Authors:** Kassa Haile Merga, Senedu Bekele Gebreegziabher, Edlawit Mesfine Getachew, Manual Kassaye Sibhatu, Hassen Mohammed Beshir, Tsegaye Hailu Kumssa, Akililu Alemu Ashuro, Endawoke Amsalu Alemayue, Mikiyas Teferi, Desalegn Bekele Taye, Berhane Redae Meshesha, Wuletaw Chane Zewude, Mulatu Biru Shagre

**Affiliations:** 1 Armauer Hansen Research Institute, Ethiopia; 2 Jhpiego Ethiopia, Johns Hopkins University Affiliate, Ethiopia; 3 Ministry of Health (MoH), Ethiopia; 4 St. Paul’s Hospital Millennium Medical College, Ethiopia; 5 Department of Health Sciences, Child and Family Health, Lund University, Lund, Sweden

**Keywords:** surgical care, surgical capacity, safe surgery, health facility, cross-sectional, Ethiopia

## Abstract

**Background:**

Surgical capacity is critical for ensuring optimum access to safe, affordable, and timely emergency and essential surgical care (EESC) in low- and middle-income countries (LMICs) like Ethiopia. A five-year strategic plan has been implemented during 2016–2020 in Ethiopia to improve surgical capacity.

**Objectives:**

This study aims to evaluate the impact of the five-year strategy in surgical capacity in the country.

**Methods:**

A cross sectional survey was conducted in 172 health care facilities in Ethiopia from December 30, 2020, to June 10, 2021. Descriptive statistical analysis was done using STATA statistical software Version 15.

**Findings:**

A total of 2,312 surgical workforces were available and, the surgical workforce to population ratio ranged from 1.13:100,000 for public specialized hospitals to 10.8:100,000 for health centre operation room (OR) blocks. Surgical bed to population ratio was 0.03:1000 population, and the average numbers of OR tables per facility were 34. Nearly 25% and 10% of OR tables were not functional in public primary hospitals and private hospitals, respectively. The average surgical volume to population ratio was 189:100,000.

**Conclusions:**

Following the implementation of surgical care strategy, the surgical workforce density has increased. However, the study revealed that there is still a huge unmet gap in surgical capacity. The improvement in surgical volume is very low compared to the increment in the surgical workforce density. In addition to the investment being made to build surgical capacity, emphasis needs to be put on surgical system design and strengthening surgical system efficiency.

## Introduction

Lack of access to safe, affordable and timely emergency and essential surgical care (EESC) is a major gap to ensure universal health coverage (UHC) in low- and middle-income countries (LMICs). Consequently, this problem poses to an increased mortality, morbidity and avoidable disability and deformity [1,2,3]. In order to avert these problems in LMICs and improve their capability to deliver EESC, enhancing the capacity of health facilities with surgical workforce, infrastructure, medications, equipment and supplies is crucial [1,4,5].

According to the Lancet Commission on Global Surgery (LCoGS), capacity, in terms of workforce and infrastructure is one of the four components to improve access to surgery. LCoGS sets targets to be achieved by 2030. Of these, the main ones are 80% coverage of essential surgical and anaesthesia services per country (within two hours access to the facility), at least 20 surgical workforce per 100,000 population, 5,000 procedures per 100,000 population annually, and 100% protection against catastrophic expenditure from out-of-pocket payments for surgical and anaesthesia care [6,7]. However, many LMICs have challenges to achieve these targets [8,9].

The Save Lives through Safe Surgery strategic plan (SaLTs) was implemented in Ethiopia for five-years, 2016–2020, intended to address the huge unmet need for basic surgical care services. The proposed strategies were well aligned with the Health Sector Transformation Plan 2 (HSTP2), recommendations of the World Health Organization (WHO) and quality strategy.

This strategy was instrumental to define and standardize the minimum care packages needed to expand EESC. The SaLTS strategy had eight strategic pillars, three of which were focused on surgical capacity (infrastructure, supplies and logistics management, and human resource development) [10,11].

Before and at the beginning of the strategic period there was a critical gap in the health workforce in general and the surgical workforce in particular [12]. The surgery, anaesthesia, and obstetrics workforce (SAO) density was 0.35:100,000 population and the surgical volume was 43:100,000 population [11].

As to our knowledge, there is limited evidence which showed the surgical capacity of the Ethiopia comparing with the recommendations of WHO and the LCoGS. Therefore, the aim of this study was to assess the surgical capacity and its effect on surgical volume in different levels of health care strata in Ethiopia after the implementation of a five-year strategic plan.

## Methods

### Study design

Health facility based cross-sectional study with retrospective data review was conducted from December 30, 2020 to June 10, 2021 to assess the surgical capacity and its effect in surgical volume after implementing the five-year SaLTS I strategy in Ethiopia.

### Study setting

Ethiopia is the second most populous country of Africa and ranks 12^th^ in the world with a population of about 101 million in 2020 [13]. Primary Health care units (PHCUs (17,550 health posts and 3,735 health centres) are the main sites of primary health care services, especially for rural communities in Ethiopia. Hospital-based services are provided by 353 hospitals that are categorized into primary, general and specialized hospitals. A total of 282 government health facilities and 45 private hospitals were providing surgical care in the country during the study period. This program evaluation was conducted in 172 sample public and private health facilities. Ministry of Health of Ethiopia implemented a five-year surgical care plan to improve access to safe and affordable emergency and essential surgical care.

### Sampling procedure and sample size

A multi-stage stratified random sampling method was used to select study sites (public and private health care facilities). Two hundred eighty two government hospitals were providing emergency and essential surgical care in Ethiopia. Of these 26 were referral hospitals, 75 were general hospitals and 181 were primary hospitals. The required sample size for the study was estimated using a single population proportion formula for a finite population with 5% margin of error and 95% level of confidence (P = 0.5). The sample size was determined to be 163 public hospitals and with stratification, 105 primary hospitals, 43 general hospitals, and 15 referral hospitals were included. Among the 45 private health facilities providing surgical care in Ethiopia, 40 of them were sampled. This makes 203 total study sites. We randomly selected the health facilities from each stratum. However, because of security issues in Ethiopia we could not access all health facilities that were selected randomly, and we decided to replace some health facilities which were convenient assuming that hospitals in the same strata are homogeneous. Accordingly, a total of 172 study sites take part in the study.

### Data collection procedures and tools

We used Surgical Assessment Tool (SAT), jointly developed by the Harvard Program in Global Surgery and Social Change (PGSSC) and the WHO. The tool was adopted and validated in the context of Ethiopian health facilities [10]. The assessment consisted of hospital visit and interviews with hospital directors and surgical and anaesthesia care providers using the tool designed to assess the domains of – infrastructure, workforce (focused on specialist surgical, obstetrics, and anaesthetics care providers), Medicine, equipment, and supplies.

Data collectors received adequate training about the entire process of data collection including quality control measures (such as completeness, correctness, concordance), piloted and synchronizing, and archiving the data with Research Electronic Data Capture (REDCap). Data collection was focused mainly on the number of surgical workforce by profession (Surgeon, Anaesthetist, Obstetrician, Integrated Emergency Surgical Officers (IESO), and Nurse Anaesthetists) at primary, general and specialized Hospitals, Infrastructure, including surgical beds and operation rooms (ORs), tables, intensive care units (ICUs), medical equipment and supplies, and surgical volume. The collected data were uploaded to the REDCap.

### Data management and analysis

Data cleaned, entered, and then exported into STATA statistical software Version 15 for further statistical analysis. Additional data cleaning and consistency checks were done to detect outliers and inconsistent variables. Descriptive statistics: Percentage, frequency, and visual graphs were used.

### Ethical consideration

Ethical clearance was obtained from the Armauer Hansen Research Institute (AHRI) ethical review committee. Letter of support to conduct the evaluation was obtained from the Ministry of health (MOH)-Ethiopia. Additionally, letters of support and permissions were obtained from the local health offices authorities to conduct data review at the selected health facilities.

### Role of the funding source

This study was funded by the MOH-Ethiopia. Jhpiego Ethiopia gave additional support to complete the data collection and write up workshop. Experts from MOH-Ethiopia, AHRI and Jhpiego Ethiopia lead the design and implementation of the project.

## Results

### Health facilities

This study included a total of 172 health facilities (81.4% public health facilities and 18.6% private health facilities). Of the public health facilities 44.8% and 22.1% were primary and general hospitals, respectively (Table 1).

**Table 1:** Level and number of evaluated health care facilities from December 30, 2020 to June 10, 2021, Ethiopia.


HEALTH CARE FACILITY LEVEL	NUMBER	PERCENTAGE

Public Specialized Hospitals	16	9.30

Public General Hospitals	38	22.09

Public Primary Hospitals	77	44.76

Health Centre OR Blocks	9	5.23

Private Hospital	32	18.60

Total	172	100


### Surgical workforce

Health facilities had a total of 2,312 surgical workforce. Of those, 51.14% and 22.98% of Surgeons (General, neurosurgeons and orthopaedic surgeons), 48.27% and 11.67% of anaesthesia care providers (anaesthesiologists or anaesthetists) and 49.4% and 23.5% of obstetricians were working at public specialized hospitals and private hospitals, respectively. Fifty-nine percent and 30.2% of Integrated Emergency Surgical Officers (IESO) were available in public primary and general hospitals, respectively (Table 2).

**Table 2:** Number of surgical workforce available in the evaluated health care facilities from December 30, 2020 to June 10, 2021, disaggregated by level of health care, Ethiopia.


HOSPITAL STAFF	NUMBER OF AVAILABLE HUMAN RESOURCE					

	SPECIALIZED HOSPITAL N = 16	GENERALIZED HOSPITAL N = 38	PRIMARY HOSPITAL N = 77	HEALTH CENTRE OR BLOCK N = 9	PRIVATE HOSPITAL N = 32	TOTAL

Surgeons (General, neurosurgeons and orthopaedic surgeons)	336 (51.14%)	123 (10.72%)	47 (7.15%)	0.00%	151 (22.98%)	657 (100%)

Anaesthesiologists or anaesthesia care providers	364 (48.27%)	157 (20.82%)	126 (16.71%)	19 (2.52%)	88 (11.67%)	754 (100%)

Obstetrician	165 (49.40%)	76 (22.75%)	16(4.79%)	0.00%	77 (23.05%)	334 (100%)

IESO	10 (3.02%)	100 (30.21%)	194 (58.61%)	18 (5.44%)	9 (2.72%)	331 (100%)

Nurse anaesthetists	14 (5.93%)	94 (39.83%)	79 (33.47%)	2 (0.85%)	47 (19.92%)	236 (100%)

Total surgical work force	889 (38.45%)	550 (23.79%)	462 (19.98%)	39 (1.69%)	372 (16.09%)	2312 (100%)


### Surgical work force to population ratio

The ratio of the surgical workforce to population served ranges from 1.31:100,000 population for public specialized hospital to 10.8:100,000 population for the health center OR blocks (Table 3).

**Table 3:** Ratio of surgical workforce per 100,000 populations served from December 30, 2020 to June 10, 2021, disaggregated by level of care, Ethiopia.


HEALTH CARE FACILITY LEVEL	NUMBER OF EVALUATED HEALTH FACILITIES	CATCHMENT POPULATION TO BE SERVED	*NUMBER OF SURGICAL WORKFORCES	SURGICAL WORKFORCE RATIO PER 100,000 POPULATION SERVED

^*^Health centre OR block	9	360,000	39	10.8:100,000 population

Public primary hospital	77	6,160,000	462	7.5:100,000 population

Public general hospital	38	47,500,000	550	1.2:100,000 population

Public specialized hospital	16	68,000,000	889	1.3:100,000 population


*Note*: Private hospitals do not have specific cathment population.

### Surgical beds

The total numbers of hospital beds available in the included hospitals were 18,418, 21% of which were surgical and 18% obstetrics care hospital beds. In the 172 health facilities, there were 232 and 398 functional operation rooms for minor and major surgeries, respectively.

Specialized hospitals had 1,375 surgical beds (trauma, general surgery and orthopaedics) and 1,089 obstetric/gynaecologic beds. Public generalized hospitals had 1,139 surgical beds and 937 gynaecologic/obstetric beds. Private hospitals had 674 surgical beds and 380 gynaecologic/obstetric beds. On the other hand, 99 of functioning operating rooms for major surgical procedures were illustrated in public primary hospitals while private hospitals and public specialized hospitals had 89 and 90 functioning major operating rooms (Table 4).

**Table 4:** Number of available surgical beds from December 30, 2020 to June 10, 2021, disaggregated by health care facility level, Ethiopia.


HOSPITAL BEDS	NUMBER OF AVAILABLE SURGICAL BEDS BY HEALTH CARE FACILITY LEVEL					

	SPECIALIZED HOSPITAL	GENERALIZED HOSPITAL	PRIMARY HOSPITAL	HEALTH CENTRE OR BLOCK	PRIVATE HOSPITAL	TOTAL

	7,330	4,864	3,699	159	2,366	18,418

Surgical beds (Trauma, General Surgery and Orthopaedics)	1,375	1139	726	36	674	3,950

Obstetric and gynaecologic beds	1,089	937	843	116	380	3,365

Functioning operating rooms (Minor surgery)	71	42	77	8	34	232

Functioning operating rooms (Major surgery)	110	90	99	10	89	398


### Ratio of surgical beds to the population served

The total number of surgical beds in the included health facilities ranges from 36 in health centres OR blocks to 1,375 in specialized hospitals. The ratio of surgical beds to the population served is: 1:10,000, in Health Centre OR block, 1:8484, in Public Primary Hospital, 1:41703, in Public General Hospital, and 1:49454 in Public Specialized Hospital (Table 5).

**Table 5:** Ratio of surgical beds to total population served disaggregated by level of health care, Ethiopia, December 30, 2020 to June 10, 2021.


HEALTH CARE FACILITY LEVEL	CATCHMENT POPULATION	NUMBER OF EVALUATED HEALTH CARE FACILITIES	TOTAL NUMBER OF SURGICAL BEDS	SURGICAL BEDS TO POPULATION RATIO

Public Primary Hospital	6,160,000	77	726	1:8484

Public General Hospital	47,500,000	38	1139	1:41703

Public Specialized Hospital	68,000,000	16	1375	1:49454

Health Centre OR block	360,000	9	36	1:10,000


*Note*: Private hospitals do not have specific cathment population.

### Operating room (OR) tables functionality

The average numbers of operating tables (OTs) in primary hospitals were 2.26 and 75.28% were functional. The average numbers of OTs in private hospitals were 3.6 and 89.65% were functional. The average numbers of OTs in the general and specialized hospitals were 3.28 and 9.9, and of these 90.4% and 84.8% were functional, respectively (Table 6).

**Table 6:** Proportion of functional operating theatre tables in a 90 days interval of the study period starting from September 2020 to May 2021, disaggregated by level of health care, Ethiopia.


HEALTH CARE FACILITY LEVEL	NUMBER OF HEALTH FACILITIES EVALUATED	TOTAL NUMBER OF FUNCTIONAL ORS	TOTAL NUMBER OF OT TABLES	NUMBER OF FUNCTIONING OT TABLES	PROPORTION OF FUNCTIONING OT TABLES	AVERAGE NUMBER OF OT TABLES PER FACILITY

Public Primary Hospital	77	176	174	131	75.28%	2.26

Public General Hospital	38	132	125	113	90.40%	3.28

Public Specialized Hospital	16	181	158	134	84.81%	9.87

Health Centre OR block	9	18	15	12	80.00%	1.66

Private Hospitals	32	123	116	104	89.65%	3.63

Total	172	630	588	494	84.01%	3.42


### Reasons for operating room tables not in use

**Figure 1:**
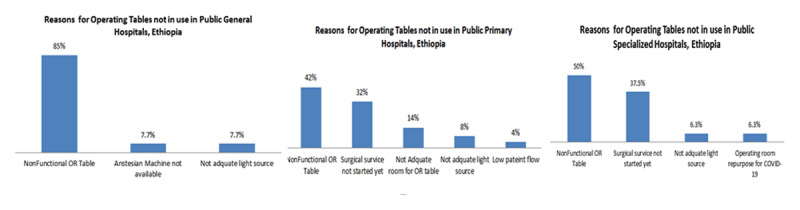
Reasons for operating room tables not in use in public primary, public general hospitals and public specialized hospitals in a 90 days interval of the study period starting from September 2020 to May 2021, Ethiopia.

The reasons for non-utilised OR tables ranged from non-functional OR tables (34.5%) to repurposing the OR table for COVID-19 treatment (3.63%), and lack of skilled professionals (3.63%). Twenty-one point eight percent of the OR tables are not functional due to the elective surgical service is not yet started in the facilities (Figure 1).

### Availability of Medical Equipment and supplies

Seventy-seven primary hospitals were assessed and 24% and 49.6% did not have adult and paediatric McGill forceps, respectively. About 35.1%, 27.3% and 24.7% of primary hospitals had no tracheostomy set, chest tube insertion instrument, and splints for arm and legs, respectively. Nearly 12% of primary hospitals did not have electrocautery and there was shortage in 66.7% of health centre OR blocks. Similarly, the facilities reported that from 64.5% to 30.6% had a shortage in electrocautery (Table 7).

**Table 7:** Availability of emergency and essential Major and minor surgical care kits, OR equipment and supplies, reporting period starting from September 2020 to May 2021, disaggregated by level of health care facilities.


EMERGENCY AND ESSENTIAL SURGICAL CARE EQUIPMENT AND SUPPLIES	LEVEL OF HEALTH CARE FACILITY														

	PRIMARY HOSPITALS (N = 77)			GENERAL HOSPITALS (N = 38)			SPECIALIZED HOSPITALS (N = 16)			HEALTH CENTRE OR BLOCKS (N = 9)			PRIVATE HOSPITALS (N = 32)		
				
	ABSENT N (%)	AVAILABLE WITH SHORTAGE N (%)	FULLY AVAILABLE N (%)	ABSENT N (%)	AVAILABLE WITH SHORTAGE N (%)	FULLY AVAILABLE N (%)	ABSENT N (%)	AVAILABLE WITH SHORTAGE N (%)	FULLY AVAILABLE N (%)	ABSENT N (%)	AVAILABLE WITH SHORTAGE N (%)	FULLY AVAILABLE N (%)	ABSENT N (%)	AVAILABLE WITH SHORTAGE N (%)	FULLY AVAILABLE N (%)

Scalpel with blades	1(1.30%)	17(22.08%)	59(76.62%)	0	2(5.41%)	35(94.59%)	1(6.25%)	1(6.25%)	14(87.50%)	0	2(22.22%)	7(77.78%)	0	1(3.23%)	30(96.77%)

Retractors	0	15(19.48%)	62(80.52%)	0	1(2.7%)	36(97.3%)	0	1(6.25%)	15(93.75%)	0	0	9(100%)	0	1(3.23%)	30(96.77%)

Scissors	0	18(23.38%)	59(76.62%)	0	7(18.92%)	30(81.08%)	1(6.25%)	3(18.75%)	12(75%)	0	1(11.11%)	8(88.89%)	0	1(3.23%)	30(96.77%)

Tissue forceps	0	16(20.78%)	61(79.22%)	0	7(18.92%)	30(81.08%)	1(6.25%)	2(12.50%)	13(81.25%)	0	1(11.11%)	8(88.89%)	0	1(3.23%)	30(96.77%)

Needle holder	0	19(24.68%)	58(75.32%)	0	6(16.22%)	31(83.78%)	1(6.25%)	2(12.50%)	13(81.25%)	0	0	9(100%)	0	1(3.23%)	30(96.77%)

Adult McGill forceps	24(32%)	17(22.67%)	34(45.33%)	2(5.41%)	5(13.51%)	30(81.08%)	0	4(26.67%)	11(73.33%)	5(55.56%)	2(22.22%)	2(22.22%)	1(3.23%)		

Paediatric McGill forceps	38(49.35%)	15(19.48%)	24(31.17%)	13(36.11%)	4(11.11%)	19(52.78%)	1(6.67%)	5(33.33%)	9(60%)	8(88.89%)	1(11.11%)	0	4(12.90%)		

Needles & sutures	0	20(25.97%)	57(74.03%)	0	7(18.92%)	30(81.08%)	1(6.25%)	2(12.50%)	13(81.25%)	0	1(11.11%)	8(88.89%)	0	3(9.68%)	28(90.32%)

Suction pump	1(1.3%)	30(38.96%)	46(59.74%)	0	11(28.95%)	27(71.05%)	1(6.25%)	5(31.25%)	10(62.50%)	0	1(11.11%)	8(88.89%)	0		

Light source (lamp & flash light)	3(3.90%)	38(49.35%)	36(46.75%)	1(2.70%)	14(37.84%)	22(59.46%)	1(6.25%)	6(37.50%)	9(56.25%)	0	2(22.22%)	7(77.78%)	0	2(6.45%)	29(93.55%)

Tourniquet	15(19.48%)	23(29.87%)	39(50.65%)	3(8.11%)	6(16.22%)	28(75.68%)	3(18.75%)	5(31.25%)	8(50%)	2(2.22%)	2(2.22%)	5(5.56%)	0	3(9.68%)	28(90.32%)

Splints for arm, leg	19(24.68%)	23(29.87%)	35(45.45%)	12(32.43%)	3(8.11%)	22(59.46%)	3(18.75%)	5(31.25%)	8(50%)	5(55.56%)	3(33.33%)	1(11.11%)	2(6.45%)	2(6.45%)	27(87.10%)

Electrocautery	9(11.69%)	22(28.57%)	46(59.74%)	0	11(30.56%)	25(69.44%)	0	8(50%)	8(50%)	6(66.67%)	2(22.22%)	1(11.11%)	0	2(6.45%)	29(93.55%)

Chest tubes insertion equipment	21(27.27%)	28(36.36%)	28(36.36%)	5(13.51%)	8(21.62%)	24(64.86%)	3(18.75%)	3(18.75%)	10(62.50%)	9(100%)	0	0	2(6.45%)	1(3.23%)	28(90.32%)

Tracheostomy set	27(35.06%)	20(25.97%)	30(38.96%)	8(21.62%)	8(21.62%)	21(56.76%)	1(6.25%)	2(12.50%)	13(81.25%)	9(100%)	0	0	0	2(6.45%)	29(93.55%)


### Surgical Volume

The total number of surgeries in the three months of study period was 57,722. When this figure is calculated to give the annual volume of surgery, it is around 230,886 surgeries annually. This makes the average surgical volume to be 189 per 100,000 population in Ethiopia.

### Discussion

We included 172 health facilities from the primary, secondary, and tertiary levels of care, from both the public and private sectors. The study was done to evaluate a five years strategic plan which was implemented to improve surgical care.

The availability of specialist surgeons, gynaecologists, and anaesthesiologist decreases when we go through from the higher to the lower level of health care in Ethiopia, and the reverse is for IESO. This might be one of the reasons for patient referrals to specialized care, and in line with the government investment. Similarly, the lower health care facilities have limited infrastructure: surgical beds, equipment, and supplies compared to the higher level of care for the same reason.

The private health facilities are well staffed with surgeons and obstetricians next to the public specialized hospitals, whereas health centre OR blocks are exclusively equipped with IESO and Anaesthesia providers. The surgical workforce ratio per 100,000 populations is very low compared to the target sated by the LCoGS, but it is 10.8:100,000 populations which is substantially higher for health centre OR blocks than primary and general hospitals. This might be due to the fact that health centre OR blocks were established only in urban settings with the expectation to serve one health centre OR block for 40,000 catchment area population (HSTP 2), which gives an advantage of serving less catchment area population when compared to the health facilities that exist outside of the urban setting. However, the Lancet Commission target for 2030 is 20 per 100,000 population, which shows that the current Ethiopian status is far behind the target [6]. This finding is in line with cross-sectional studies conducted previously in Ethiopia and Liberia [8,14]. Therefore, interventions including strengthening of the existing task shifting approach and monitoring of its effectiveness are among the viable ways to scale up the surgical workforce. Nevertheless, the specialized heath workforce capacity was high in the higher health care facilities (7% vs 51%) in primary and tertiary hospitals which is much higher than the finding in the study in rural India (13.8% vs 4.7%), respectively [9].

The average numbers of hospital beds available at all levels were in the range of the Ethiopian standard. Surgical, gynaecology, and obstetrics beds account for 40% of the total hospital beds. The other finding is that several OR tables are non-functional, 25% of them in primary hospitals while about 10% in private hospitals. Similarly, several operation rooms are not in use and the main reason was non-functional OR table followed by lack of elective surgery service.

The availability of surgical beds is 0.03 surgical beds:1000 people; this is markedly lower than the total number of 0.3 hospital beds:1000 population ratio in Ethiopia in 2016 [15]. In contrast, the availability of surgical beds reported in this study is also lower than that in a study conducted in South Africa [12]. Possible reason include the Gross Domestic Product (GDP) of the South Africa is better than our study setting, which might have attributed for availability of medical equipment. In this study, the average number of operation tables per facility was 3.4, which is very low to achieve the Lancet Commission target of 5000 procedures per 100,000 populations.

In this study, health care facilities are lacking a variety of surgical items which is creating a barrier to the ability of the health system to deliver health services to patients. The shortage of the medical equipment is higher in primary hospitals than specialized hospitals. The possible reason might be poor maintenance and repairs as well as limited financial resources are responsible for the shortages. Therefore, proper management is required to develop and implement procurement, maintenance and quality control plans.

This study revealed that the surgical capacity in Ethiopia is far behind from the targets of the LCoGs. However, the surgical workforce has increased by nearly fourteen-fold than it has been four years before (from 0.35 to 5.19:100,000 population). This may be due to the accelerated training of surgical workforce in Ethiopia including IESO and nurse anaesthetists [11]. The health care facilities that can provide EESC have also increased. All this is intended to enhance access for surgical and increase annual surgical volume. Regardless, even with this progressive increment of capacity for surgical care, the surgical volume didn’t show improvement that can match with the growth in capacity (Annual estimated volume increased from around 200,000 to 347,976 after five years or 43:100,000 to 261.5:100,000 population) [10]. This might be due to deficiency in system design and management intending to improve efficiency in surgical care.

As like any cross-sectional studies, this study also has a number of limitations. Firstly, due to the security situation we were not able to include all the health facilities selected for the study, although we did replacement for some health facilities. This may put nationwide representation of the study under the question. Moreover, as this study was conducted in the era of the COVID-19 pandemic, it might have affected the activities of health care facilities. Nevertheless, this study is believed to provide a crucial insight about surgical capacity and related challenges at different levels of health care delivery in Ethiopia, thus, the result could be used as a base to develop surgical capacity improvement strategies.

## Conclusions and recommendations

Overall, this surgical capacity assessment revealed critical gaps in all parameters included in the assessment. However, there is an increment of health care facilities and the surgical workforce in the past five years that can provide essential and emergency surgical care. Nevertheless, surgical volume did not match with the capacity improvement for surgical care. This implies, there is a need to give due emphasis in system design and management to improve efficiency and effectiveness in surgical care parallel to capacity building. The findings from this assessment can be used as a crucial input for the development of the country’s strategic plan of Safe Surgery intervention and can guide interventions to strengthen the surgical system in Ethiopia and other similar settings with resource constraints.
